# Use of an Atrial Lead with Very Short Tip-To-Ring Spacing Avoids Oversensing of Far-Field R-Wave

**DOI:** 10.1371/journal.pone.0038277

**Published:** 2012-06-22

**Authors:** Christof Kolb, Georg Nölker, Carsten Lennerz, Hansmartin Jetter, Verena Semmler, Klaus Pürner, Klaus-Jürgen Gutleben, Tilko Reents, Klaus Lang, Ulrich Lotze, for the AVOID-FFS Investigators

**Affiliations:** 1 Deutsches Herzzentrum und 1. Medizinische Klinik rechts der Isar, Faculty of Medicine, Technische Universität München, Munich, Germany; 2 Klinikum Coburg, II. Medizinische Klinik, Coburg, Germany; 3 Herzzentrum Nordrhein-Westfalen, Ruhr-Universität Bochum, Bad Oeynhausen, Germany; 4 Klinikum Memmingen, Medizinische Klinik I, Memmingen, Germany; 5 Kreisklinik Ebersberg, Abteilung Innere Medizin, Ebersberg, Germany; 6 Kreiskrankenhaus Ottweiler, Abteilung für Innere Medizin, Ottweiler, Germany; 7 DRK-Krankenhaus Sondershausen, Abteilung Innere Medizin, Sondershausen, Germany; Texas Heart Institute, United States of America

## Abstract

**Objective:**

The AVOID-FFS (**Avoid**ance of **F**ar-**F**ield R-wave **S**ensing) study aimed to investigate whether an atrial lead with a very short tip-to-ring spacing without optimization of pacemaker settings shows equally low incidence of far-field R-wave sensing (FFS) when compared to a conventional atrial lead in combination with optimization of the programming.

**Methods:**

Patients receiving a dual chamber pacemaker were randomly assigned to receive an atrial lead with a tip-to-ring spacing of 1.1 mm or a lead with a conventional tip-to-ring spacing of 10 mm. Postventricular atrial blanking (PVAB) was programmed to the shortest possible value of 60 ms in the study group, and to an individually determined optimized value in the control group. Atrial sensing threshold was programmed to 0.3 mV in both groups. False positive mode switch caused by FFS was evaluated at one and three months post implantation.

**Results:**

A total of 204 patients (121 male; age 73±10 years) were included in the study. False positive mode switch caused by FFS was detected in one (1%) patient of the study group and two (2%) patients of the control group (p = 0.62).

**Conclusion:**

The use of an atrial electrode with a very short tip-to-ring spacing avoids inappropriate mode switch caused by FFS without the need for individual PVAB optimization.

**Trial Registration:**

ClinicalTrials.gov NCT00512915

## Introduction

Diagnostic features of modern pacemakers are frequently used in monitoring the clinical status of patients including the potential occurrence of atrial tachyarrhythmias [1]–[6]. In this setting, derived data logs displaying mode switch event frequency and duration provide assistance when deciding whether or not antiarrhythmic or anticoagulation treatment is indicated. However, this decision making is highly dependant on the reliability of the data provided by the device. Far-field R-wave sensing (FFS) has been identified as the most common cause of inappropriate mode switch in dual chamber pacemakers [7], [8]. False positive mode switches can result in a loss of atrioventricular synchrony in atrioventricular block and can cause impaired hemodynamics, potential proarrhythmic effects, and pacemaker syndrome. It may also lead to commencement of unnecessary antiarrhythmic medication, interventional therapy or anticoagulation.

Current strategies to avoid inappropriate mode switch from DDD(R) to DDI(R) mode and to optimize the quality of stored data include the programming of a reduced atrial sensitivity [9], [10] or the individual adjustment of the postventricular atrial blanking period (PAVB) [8], [10]–[12]. Each of these strategies encounters its own problems. Reducing the atrial sensitivity runs the risk of undersensing during atrial tachyarrhythmias [13], [14] and can therefore end in an underestimation of the atrial tachyarrhythmia burden. Adjusting the PVAB – although highly effective in avoiding inappropriate mode switch [8], [10]–[12] – encounters some extra time for individual optimization of the parameter in follow-up visits and it is not devoid of tachyarrhythmia misclassification as a 2∶1 lock-in of atrial signals may occur with atrial flutter [15].

Recent studies have shown that reducing the inter-electrode tip-to-ring spacing of atrial bipolar leads reduces the amplitude of far-field R-wave and consequently the occurrence of inappropriate mode switches [16], [17]. With the availability of new leads providing a very short tip-to-ring distance of 1.1 mm instead of traditional 10 mm spacing, FFS may be further reduced [18]–[20] and individual adjustment of pacemaker settings may no longer necessary to avoid FFS.

Therefore, the aim of the prospective, randomized AVOID-FFS study was to compare the incidence of inappropriate mode switch due to FFS between pacemaker systems supplied with an atrial lead of a very short bipole electrode spacing (1.1 mm) to those with a standard spacing (10 mm). The comparison was done under the precondition that in pacemaker systems with the very short atrial inter-electrode spacing the PVAB was programmed at 60 ms without any further adjustment, whereas in pacemaker systems using atrial electrodes with conventional bipole spacing the PVAB was individually optimized according to an additional test.

## Methods

The protocol for this trial and supporting CONSORT checklist are available as supporting information; see Checklist S1 and Protocol S1. A total of 10 centers participated in the AVOID-FFS study between February 2007 and May 2009 (ClinicalTrials.gov identifier: NCT00512915). Patients with indication for dual chamber pacing (according to national or international guidelines) and receiving an Identity (ADx) DR or Victory DR pacemaker (St. Jude Medical, Sylmar, CA, USA) were eligible for the study. They were randomly assigned (1∶1 allocation in blocks of four in centrally generated sealed envelopes) to receive a novel atrial lead with a very short tip-to-ring distance (OptiSense™ 1699T or 1999T, both from St. Jude Medical, Sylmar, CA, USA) or a conventional atrial lead (Tendril™ 1388T, 1688T, 1788T or 1888T, all from St. Jude Medical, Sylmar, CA, USA). All of these steroid-eluting, active fixation leads are characterized by practically identical values for cathode and anode surface and amount of helix extension, but the leads differ in the spacing between the distal tip electrode and the proximal ring electrode which is 1.1 mm for the OptiSense™ leads and 10 mm for the other leads.

All leads were implanted according to standard protocols and the atrial implant site was left at the discretion of the implanting physician.

All patients gave their written informed consent for the study procedure, and the trial was approved by the ethics committee of the Technische Universität München, Munich, Germany (project number 1694/07) and it was conducted under the conditions laid out in the declaration of Helsinki.

Patients with persistent or permanent atrial tachyarrhythmias, patients undergoing a pacemaker replacement or those not available for follow-up visits as well as minors were excluded from the study. Other exclusion criteria were pregnancy, mental disability, history of cardiac surgery or myocardial infarction within 4 weeks prior to enrolment or planned cardiac surgery within the next 3 months after randomization.

### Study Protocol

In both groups pacemakers were programmed to the DDD(R) mode and atrioventricular delays were optimized (mere prolongation or use of the hysteresis algorithm) to allow intrinsic atrioventricular conduction if present. The atrial sensitivity level was set to 0.3 mV and mode switch was enabled for atrial rates greater than 180 bpm according to the filtered atrial rate interval used in St. Jude Medical pacemakers. The trigger to store an electrogram in the pacemaker was activated for mode switch and all other triggers were turned off.

In the control group PVAB was optimized according to a far-field R-wave sensing test. For this test the atrial sensitivity was temporarily set to 0.1 mV and PVAB to the lowest setting of 60 ms. FFS was provoked during ventricular stimulation. The optimized PVAB setting was defined as the interval between the ventricular pacing marker and the detection of a far-field R-wave signal (corresponding to an atrial event sensed in the refractory period) plus 25 ms. If no far-field R-wave signal was detected by the pacemaker, the optimized PVAB was set to 85 ms. In the study group receiving the lead with the very short bipole distance this optimizing test was not applied and the PVAB was fixedly programmed to a value of 60 ms. Additionally, in both groups sensing threshold, pacing threshold, and lead impedance were determined at discharge, one month and three months after pacemaker implantation.

The primary endpoint of the study was the occurrence of inappropriate mode switch due to FFS assessed by stored electrograms of the pacemaker within the first 3 months after implantation.

The secondary endpoints of the study were fluoroscopy time and cut-to-suture time during implantation, lead parameters during follow-up, the influence of the atrial lead position and the ventricular pacing percentage on the occurrence of inappropriate mode switch caused by FFS, and the occurrence of symptomatic 2∶1 lock-in of atrial flutter.

### Inappropriate Mode Switch Due to FFS

Atrial far-field R-wave signals were defined as atrial signals that were unrelated to an intrinsic atrial activation but were related to a ventricular depolarization. These far-field signals differ in size, morphology and potentially in polarity from the atrial near-field signal. In contrast to supraventricular premature beats there is a constant coupling interval between a ventricular event and the atrial far-field signal for each form of ventricular depolarization (ventricular paced beat, intrinsic atrioventricular conduction, ventricular premature beats of same origin).

**Figure 1:**
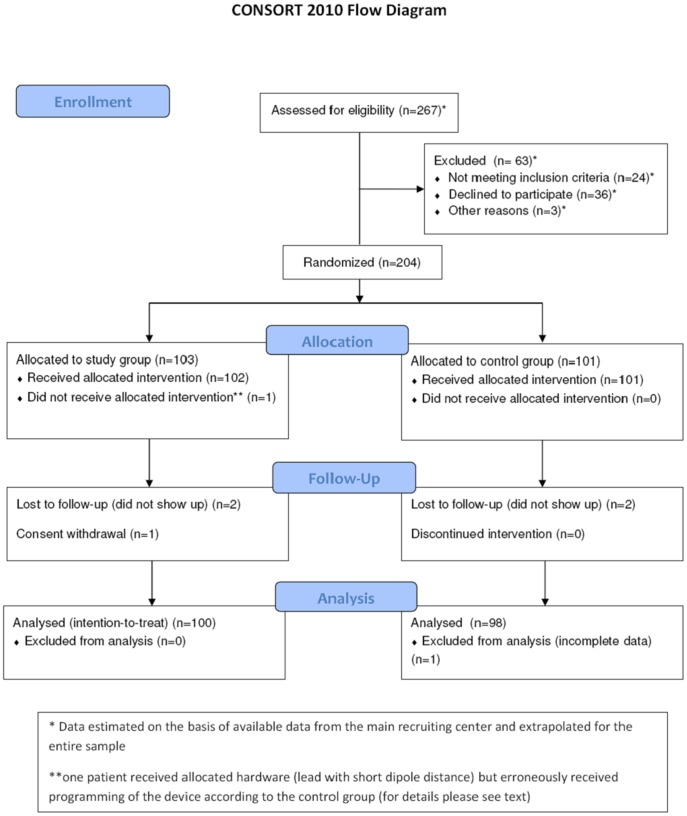
Study flow-chart.

### Statistical Analysis and Sample Size Calculation

Based on previous published results the incidence of inappropriate mode switch due to FFS is 5% among patients with individual optimization of the PVAB [8]. Given a significance level of 5%, a power of 80%, and the lower limit of the observed 95% confidence interval exceeding −10%, a total of 75 complete data sets per group were calculated to determine non-inferiority. Including drop outs of 10%, a total of 168 patients (84 per group) were planned to be enrolled.

Statistical analyses were performed using the software SAS 9.2 for windows (SAS Institute Inc., Cary, NC, USA.). Continuous data are presented as mean ± standard deviation, and comparisons between the groups were carried out using the Student’s t-test or the Wilcoxon test, where appropriate. Categorical variables are presented as frequencies and percentages and comparisons between the groups were performed using the Chi-square test or Fisher’s exact test, where appropriate. P-values <0.05 were considered statistically significant. Data were evaluated on an intention-to-treat basis.

## Results

### Study Population

A total of 204 patients (121 (59%) male, 83 (41%) female, mean age 73±10 years) were included in the study; 103 patients were randomly assigned to the study group and 101 to the control group, respectively. Complete data sets for the primary endpoint were available for 100 patients of the study group and 98 patients of the control group. Further information on recruitment and study conduct are given in Fig. 1. Baseline characteristics did not differ between the two groups and are shown in Table 1.

**Table 1:** Baseline characteristics of the patients.

	Study group	Control group	*P*-value
	(n = 103)	(n = 101)	
**Age ± SD [years]**	73±9	74±10	0.33
			
**Male n (%)**	61 (60)	60 (60)	0.99
**Pacemaker indication n (%)**			
Sinus node dysfunction	43 (42)	38 (38)	0.62
Atrio-ventricular block	38 (37)	46 (45)	
Binodal disease	20 (19)	15 (15)	
Other	2 (2)	2 (2)	
**History of atrial fibrillation** **n (%)**	34 (33)	39 (39)	0.48
**Atrial lead placement n (%)**			
Lateral wall*	41 (40)	44 (43)	0.53
Right atrial appendage*	49 (47)	38 (38)	
Other*	13 (13)	19 (19)	
**Fluoroscopy time ± SD [s]**	320±315	355±427	0.94
**Procedure time ± SD [min]**	61±20	62±19	0.65

* According to the implanter’s judgment.

### Primary Endpoint

**Figure 2:**
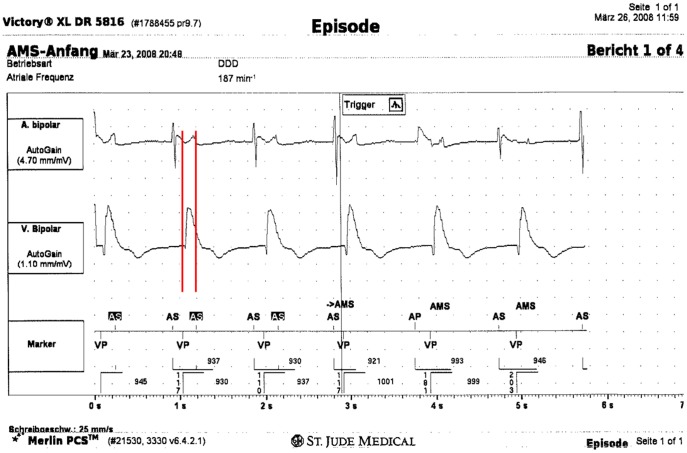
Inappropriate mode switch due to FFS: Stored episode depicting inappropriate mode switch due to FFS from the control group. Despite optimized PVAB of 140 ms inappropriate mode switch occurs because the coupling interval of the far-field R-wave is 160 ms. First line: bipolar atrial electrogram, shows bipolar atrial, second line: bipolar ventricular electrogram, bottom line: marker channel with AMS = mode switch; AP = atrial pacing; AS = atrial sensed event; AS on black background = atrial sensed event in refractory period; VP = ventricular pacing.

**Figure 3:**
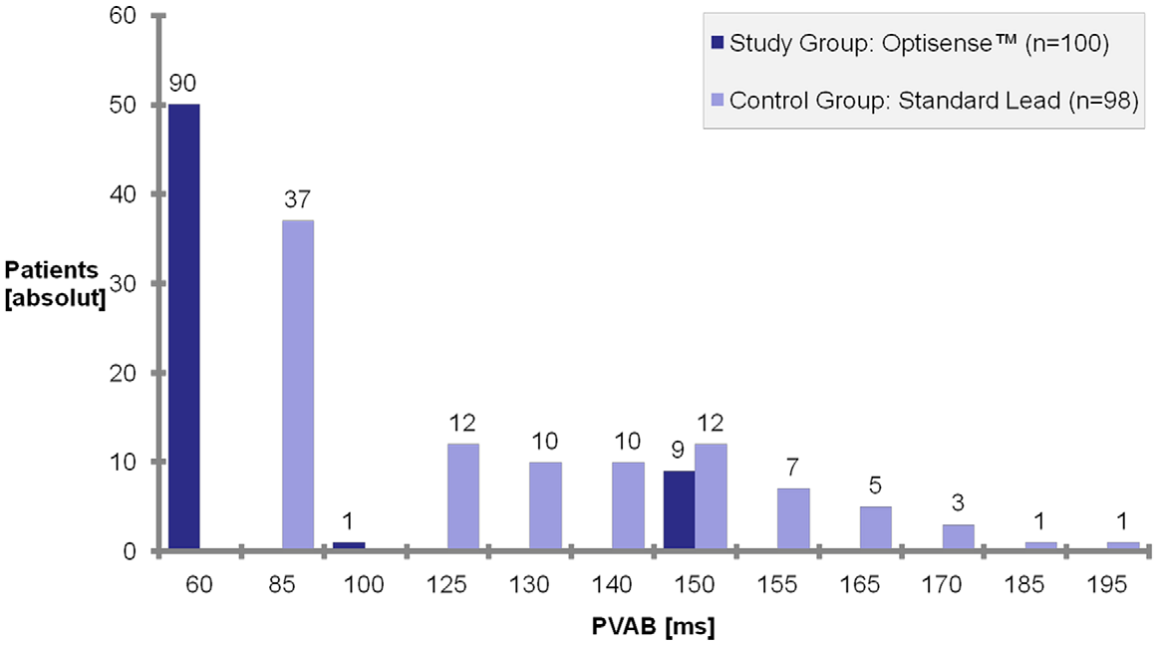
Distribution of programmed PVAB during follow-up by randomization groups. Nine patients of the study group initially received a PVAB that was erroneously programmed to a value other than 60 ms which was corrected after one month in eight of the patients and remained prolonged in one patient. None of these patients had inappropriate mode switch due to FFS. One patient of the study group patient exhibited inappropriate mode switch due to FFS and the PVAB was then individually optimized (150 ms). For the control group PVAB is shown as determined to be optimal at discharge. In case of changes of the PVAB programming during the follow-up, the longest programmed PVAB for both groups are displayed.

The primary endpoint of inappropriate mode switch due to FFS (Fig. 2) was detected in 1/100 (1%) patient of the study group and in 2/98 (2%) patients of the control group (p = 0.62). For this comparison the median or mean value (range) of the PVAB were 60 ms or 68 ms (60–150 ms) for the study group and 128 ms or 121 ms (85 to 195 ms) for the control group (p<0.005). Due to erroneous programming the PVAB exceeded the value of 60 ms, which was specified in the trial protocol, in nine patients of the study group. This false PVAB setting was corrected in eight out of nine individuals at the one month follow-up visit. In one patient of the study group, the PVAB was individually optimized and prolonged after the occurrence of inappropriate mode switch at a setting of 60 ms. The distribution of applied PVAB is summarized in Fig. 3.

The 95% confidence interval for the difference between the two groups in terms of rate of inappropriate mode switch is (−4%, 2%). This confidence interval is above the non-inferiority margin, therefore non-inferiority of the study group can be concluded.

### Secondary Endpoints

As shown in the baseline characteristics (Table 1), fluoroscopy time and procedure time did not differ between the two groups. Furthermore, all leads could be successfully placed according to the randomization. At discharge, P-wave amplitudes of OptiSense™ electrodes were lower than those of conventional atrial leads. This difference remained constant over the three months follow-up period. Atrial pacing thresholds were significantly lower in the OptiSense™ group during the whole study duration and lead impedances were constantly higher in the OptiSense™ group during the follow-up period reaching statistical significance for the measurements at discharge (Table 2). Due to the small number of patients meeting the primary endpoint a subgroup analysis could not be run in order to identify potential risk factors for the occurrence of inappropriate mode switch such as the atrial lead position or the ventricular pacing percentage. In none of the patients a symptomatic 2∶1 lock-in of atrial flutter was observed.

**Table 2:** Electrical parameters of the atrial leads.

	Study Group (n = 103)	Control Group (n = 101)	*P*-value
**P-wave amplitude [mV]**			
At discharge	2.1±1.2	2.6±1.3	0.02
At month 1	2.1±1.1	2.7±1.3	<0.001
At month 3	2.1±1.2	2.5±1.3	0.01
**Atrial pacing threshold [V] @ 0.4 ms**			
At discharge	0.57±0.25	0.64±0.26	<0.01
At month 1	0.60±0.23	0.72±0.27	<0.001
At month 3	0.59±0.18	0.72±0.21	<0.001
**Atrial lead impedance [**Ω**]**			
At discharge	408±112	343±74	<0.001
At month 1	363±67	336±67	0.01
At month 3	355±60	337±62	0.04

## Discussion

The main results of the present randomized multicenter study on the efficacy of very short tip-to-ring atrial electrodes to avoid FFS compared to conventional atrial leads can be summarized as follows: 1.) in both groups the incidence of false positive mode switch induced by FFS was comparably low 2.) the low incidence of inappropriate mode switch due to FFS was attained despite a fixed short PVAB setting of 60 ms in the very short tip-to-ring electrode group and through individually prolonged PVAB intervals in the control group. 3.) electrical performance of the atrial leads with very short tip-to-ring distance did not vary during the three months follow-up period, and 4.) in detail, P-wave amplitudes were smaller in the study group, but were yet in a clinically acceptable range. Furthermore, lower pacing threshold during follow-up could be seen in the study group.

The very few episodes of inappropriate mode switch in the study group (OptiSense™ lead) indirectly confirm the results of two recent investigations [18], [19] which found reduced ventricular far-field R-wave amplitudes in the atrium using the OptiSense™ electrode. Another study proved a significant decrease of inappropriate mode switch due to FFS by using a pacemaker system with an OptiSense™ lead compared to controls with standard leads which - however – settings were not optimized for the PVAB or the atrial sensing threshold [20]. In contrast to these reports, the present randomized trial is the first to compare the occurrence of inappropriate mode switch due to FFS with a group of patients who received a standard atrial electrode but underwent an individual optimization of the device programming, which should be regarded as “gold standard” in clinical practice.

The very low incidence of inappropriate mode switch caused by FFS in the OptiSense™ group - which reached the pre-defined non-inferiority margin - was shown under the precondition of no individual PVAB optimization and of programming the lowermost PVAB. Thus, it can be postulated that the theoretical downside of the short PVAB is fully compensated by the sensing properties of the novel lead which effectively reduces the far-field R-wave signal amplitudes below clinically applied sensing thresholds of the atrial channel. This effect is most likely due to the small bipole distance which diminishes the antenna function of the electrode.

The use of atrial leads with a very short tip-to-ring spacing provides several benefits to clinical practice: 1) an effective reduction of inappropriate mode switch due to FFS, 2) a time saving in follow-up visits as no more individual adjustments of the PVAB is needed and 3) a possible lowering of the likelihood of 2∶1 lock-in of atrial flutter [15] by the application of short PVAB intervals (median 60 ms versus 128 ms).

Apart from the superior performance in reducing FFS the leads with 1.1 mm bipole spacing also differ in basic electrical parameters when compared to standard electrodes. Pacing impedances were higher in the study group; pacing thresholds and sensing thresholds were significantly lower in the study group. Although statistically significant, the slightly better performance concerning the pacing threshold can hardly be interpreted as an advantage of the lead. The observation of reduced P-wave amplitudes remained stable over the follow-up period of three months (2.1 mV versus 2.6 mV on average) and is in line with previous publications [18]–[20]. Potentially, the smaller “antennal function” [16] of bipolar leads with very short tip-to-ring distances does not only lower the reception of far-field R-waves but may also diminish the reception of near-field myopotentials and therefore reduce measured P-wave amplitudes. Additionally, signals derived from leads with a very short bipole distance may differ in morphology from those derived from standard leads and it might be possible that device based filters alter these signals differently.

On balance, the improved signal-to-noise ratio of a lead with a very short tip-to-ring spacing outweigh the reduced signal amplitudes detected for the OptiSense™ leads during sinus rhythm. It remains to mention that both groups of leads, the OptiSense™ and the standard electrodes, do not differ in implantation and fluoroscopy time.

### Limitations

Although the drop-out rate was low (two patients in each group were lost to follow up and one patient withdrew his consent after 1 month) it cannot be excluded that this may have biased the study result.

The atrial lead positions were – according to general clinical practice - predominantly the atrial appendage or the lateral wall. Therefore, we cannot comment on the lead performances and potential detection of far-field R-wave signals at other sites such as the lower atrial septum for which a higher risk of FFS has been described [21], [22] or for the Bachmann bundle region for which low amplitudes of far-field R-wave signals have been reported [23].

The study was restricted to the use of St. Jude Medical pacemakers and leads. Although not tested, it seems likely that a similar result would have been obtained with other pacemakers that use a constant sensing threshold during the entire cardiac cycle. Latest generation pacemakers of different manufacturers enable sensing thresholds that vary during the cardiac cycle and which also depend on the intrinsic P-wave amplitude similar to the settings used in implantable cardioverter defibrillators. The performance of different leads concerning FFS in these dynamic settings warrants further investigation and the results of the present study may not necessarily be transferable. Nevertheless, fixed sensing thresholds still represent the most frequently applied detection mode in pacemakers and therefore reinforce the clinical applicability of the trial.

The follow-up was restricted to three months and therefore long-term effects cannot be addressed by this study. Far-field R-wave signals remained stable in their coupling intervals and amplitudes during a three months observation period in a previous study [8]. Considering the pathophysiology of far-field R-wave formation [24], we believe that our results are likely to be valid also in a mid-term follow-up provided that major atrial and ventricular remodeling or cardiac disease progression does not occur.

### Conclusions

Our data demonstrate that atrial leads characterized by a tip-to-ring spacing of 1.1 mm avoid inappropriate mode switch caused by far-field R-wave sensing without the need for PVAB optimization. The same low rate of inappropriate mode switch due to far-field R-wave sensing can be achieved with conventional leads when the pacemaker’s PVAB is individually optimized. The use of atrial bipolar leads with a very short tip-to-ring spacing shows the potential to reduce the follow-up time and may increase the validity of pacemaker diagnostic data by a short PVAB setting.

## Supporting Information

Checklist S1 **CONSORT Checklist.**
(DOC)

Protocol S1 **Trial Protocol.**
(DOC)
