# Supplementary material for: Use of an Atrial Lead with Very Short Tip-To-Ring Spacing Avoids Oversensing of Far-Field R-Wave
Source: PLoS One. 2012 Jun 22;7(6):e38277. doi: 10.1371/journal.pone.0038277 (PMC3382193; doi:10.1371/journal.pone.0038277)
Supplement: Protocol S1 — Trial Protocol. (DOC) [file pone.0038277.s002.doc]

# Avoid FFS

**Use of the Atrial Lead 1699 with**

**very short Tip-to-Ring Spacing to Avoid**

**Oversensing of Far-Field R-Wave**

**(Atriale Vorhofsonde 1699 mit sehr kurzem**

**Bipolabstand zur Vermeidung von**

**Far** **Field** **Sensing)**

# CLINICAL TRIAL PROTOCOL

Version 1.0 – 27.10.2006

**principal investigator:** Dr. med. Christof Kolb,
Deutsches Herzzentrum München
Lazarettstr. 36, 80636, phone +49 89-1218 0

**Sponsor:** St. Jude Medical GmbH, Helfmann-Park 1, 56760 Eschborn, phone 06196-7711-0

# Avoid FFS

# Approval of Protocol

Version 1.0 - 27.10.2006

The present version of the clinical trial protocol

**Use of the Atrial Lead 1699 with**

**very short Tip-to-Ring Spacing to Avoid**

**Oversensing of Far-Field R-Wave**

**Study Code: B84**

is applicable for conducting the study in a scientific correct way

Signature

Principal Investigator

(Dr. med. Christof Kolb)

place

date

Signature

Sponsor

St. Jude Medical GmbH
(Alexander Hümmer)

place

date

Contents:

[Avoid FFS 1](#__RefHeading__2_1121903583)

[CLINICAL TRIAL PROTOCOL 1](#__RefHeading__4_1121903583)

[Avoid FFS 2](#__RefHeading__6_1121903583)

[Approval of Protocol 2](#__RefHeading__8_1121903583)

[1 Introduction 1](#__RefHeading__10_1121903583)

[2 Hypothesis given 1](#__RefHeading__12_1121903583)

[3 Research Objectives 1](#__RefHeading__14_1121903583)

[4 Study Organisation 2](#__RefHeading__16_1121903583)

[5 Methodology 4](#__RefHeading__18_1121903583)

[6 Valid Implants 10](#__RefHeading__20_1121903583)

[7 Statistical consideration 10](#__RefHeading__22_1121903583)

[8 Stop criteria 10](#__RefHeading__24_1121903583)

[Ethics and legal aspects 11](#__RefHeading__26_1121903583)

[10 Contact 11](#__RefHeading__28_1121903583)

[12 Protocol Amendments 12](#__RefHeading__30_1121903583)

[13 References 12](#__RefHeading__32_1121903583)

## 1 Introduction

### 1.1 Current state of research

Far-field R-wave sensing (FFS) has been identified as the most common cause for inappropriate mode switch in dual chamber pacemakers.

Under default pacemaker settings and by using a conventional bipolar atrial lead the incidence of inappropriate mode switch is 20%-30%^1/2^. Applying a far-field R-wave sensing test and adjusting the post ventricular atrial blanking period (PVAB) can reduce the incidence of inappropriate mode switch to 10%^3^.

In recent studies a newly developed atrial lead with a short bipol distance has shown a relevant reduction on the far-field R-wave amplitude whereas the amplitude of the near field signal (p-wave) remains comparable to conventional leads.

The aim of the study is to show, that using an atrial lead with a very short tip-to-ring spacing can avoid inappropriate mode switch with neither any additional testing nor any individual adjustments of pacemaker settings (Sensing, PVAB)

## 2 Hypothesis given

The new atrial lead Tendril 1699 with a short tip-to-ring distance goes along with a reducing impact on the far-field R-wave amplitude and consequently avoids inappropriate mode switch. Thus by using the new leads with a short bipol spacing there is no need for additional far-field sensing tests and for individual adjustments of pacemaker settings

## 3 Research Objectives

### 3.1 Primary research objective

The aim is to compare the incidence of inappropriate mode switch (documented as stored IEGMs) between pacemaker systems supplied with a atrial lead with a short bipol spacing (Tendril 1699) and without applying a far-field sensing test or a prolonged PVAB to those with a conventional atrial lead (Tendril 1388, 1688 or 1788) and with an individually optimized PVAB according to the FFS-testing.

### 3.2 Secondary research objective

- Influence of the atrial lead position and the ventricular pacing percentage on the

occurrence of inappropriate mode switch

- Detection of atrial flutter in the pacemaker storage
- Occurrence of 2:1-Lock-in of atrial flutter

## 4 Study Organisation

### 4.1 Principle Investigator

Dr. med. Christof Kolb

Deutsches Herzzentrum München

Lazarettstr. 36

80636 München

phone: +49 89 12 18 0

### 4.2 Sponsor

St. Jude Medical GmbH
Helfmann-Park 1
65760 Eschborn

phone 06196-7711-0

fax 06196-7711-247

### 4.3 Acceptance as study centre

Prior to initiation of the Avoid FFS trial study centres have to provide the sponsor with the following documents:

- Signed "Investigator Agreement"
- CV (up to date) of the investigators und Co-Investigators
- Written and signed approval of the Ethic Committee

### 4.4 Responsibilities and duties

#### 4.5.1 Investigator und Co-Investigator

In the course of the Avoid FFS trial investigators and co-investigators have the following responsibilities and duties:

- Applying for ethical review of the study protocol by the Ethic Committee
- Providing information to the Ethic Committee in case of protocol amendments
- Providing information to and educating patients before recruiting
- Recruiting of patients meeting all of the inclusion criteria and none of the exclusion criteria
- Performing the planned examinations and doing the follow ups in line with the Avoid FFS protocol, filling in the requested forms or transferring the data via electronic storage media
- Reporting adverse events to the sponsors within one working day after occurrence
- Reporting serious adverse events additionally to the Ethic Committee and the public authorities without delay

#### 4.5.2 Principle Investigator

- In addition to the above mentioned tasks the principle Investigator (PI) has to evaluate in case of serious adverse events if continuing the study goes along without negative influence on safety and well-being of each participant or the total study population
- In case of potential harm the principle investigator will discontinue the Avoid FFS trial ahead of time.
- Approving the study protocol before trial start or after protocol amendments

#### Sponsor

- Selecting and activating new study centres after receipt of the required documents
- Monitoring
- Creating, analysing and maintaining a database
- Transferring paper-based data in the database
- Approving the study protocol prior to study start and after protocol amendments respectively

### 4.6 Data processing and data privacy

All collected and on forms documented data will be stored in anonymized form in electronic files and processed according to the current data privacy regulations and legal framework.

The database will be created and maintained by the sponsor of the Avoid FFS trial, the St. Jude Medical GmbH – Eschborn. After study completion and publication the centres and the sponsor have access to the data pool.

### 4.7 Monitoring Agreements

The run of the trial will be assisted by employees of St. Jude Medical GmbH with visits on-site and counselling hotline.

In terms of monitoring the following agreements are made:

On the condition of adequate supervision and under the guarantee of confidentiality the investigator will give the site managers (monitors) access to all stored follow-up data, the relevant sections of the anonymised patient´s file or the pacemaker settings and pacemaker derived data.

This Monitoring serves as a quality control and ensures the data accuracy in the Avoid FFS data base. In case of study related questions or technical issues the investigator is free to contact the Avoid FFS study managers or other employees of St. Jude Medical GmbH in accordance with usual practice.

### 4.8 Publishing control

After completing the trial a report will be compiled.

The Investigator agrees on publishing the collected data and gained findings along with St. Jude Medical. The collected study data can be utilized for giving talks at a congress or for medical publishing based on a mutually agreement among the parties.

The first publication of the study results can be done under the principle investigator´s authorship, if he has included an adequate number of participants.

After completing the trial and finishing the final report the study centres as well as St. Jude Medical will have access to the complete data pool

### 4.9 Modification of the study protocol

During the ongoing study new scientific findings, interim results and adverse events can require amendments. If it is necessary or reasonable to expand, modify or eliminate areas of interest and investigations, as detailed in the current study protocol, the relevant protocol sections will be revised and attached to all delivered study protocols after written approval by the principle investigator and the sponsor.

The investigators are in charge to report all amendments of the protocol to each of the responsible ethics committees.

### 4.10 Planned duration of study

The study is designed to include about 170 patients.

The recruiting period will last roughly 12 months.

With an expected start in October 2006 and a follow up period of a minimum of 3 months the study will end in the middle of 2008.

## 5 Methodology

The trial is designed as prospective, randomised, multicentric and multinational study

### 5.1 Study course

After giving a written informed consent for participating in the trial the patient will receive a dual chamber pacemaker according to the study protocol.

Prior to pacemaker implantation the patient will be randomly assigned to the study group with receiving a novel atrial lead with a short tip-to-ring distance (Tendril 1699) or to the control group with receiving a conventional atrial lead (Tendril 1388, 1688 or 1788)

In the study group the PVAB will be fixedly programmed at 60ms. Whereas in the control group the coupling interval will be defined according to a far-field sensing test and finally the optimized PVAB will be programmed at the coupling interval plus 25 ms. (see below: 5.4.5 far-field sensing test, as well as 5.5 pacemaker programming guidelines)

The primary endpoint will be the occurrence of an inappropriate mode switch due to FFS assessed by stored episodes of the pacemaker within the first 3 months after implantation.

In case of an inappropriate mode switch the far-field sensing test will also be applied to a patient of the study and the PVAB will be programmed at the coupling interval plus 25ms. The 3 months follow up of these patients will be continued.

### 5.2 Study population

In order to participate in the AVOID FFS trial, all of the following inclusion criteria must be met and all exclusion criteria must not be met. Patients with a pacemaker exchange are not eligible for the study.

#### 5.2.1 Inclusion criteria

- Indication for a dual chamber pacing system (according to national and international

guidelines)

- Implantation of a St. Jude Medical pacemaker: Identity (ADx) DR, Victory DR or models released later on with same functionality.
- Age ≥ 18 years

#### 5.2.2 Exclusion criteria

- Persistent or permanent atrial tachyarrhythmias
- Pacemaker exchange
- Pregnancy
- Participation in another trial investigating an implantable medical device
- No availability for follow-up visits
- Absence of the patient´s written informed consent form
- History of cardiac surgery or myocardial infarction within 4 weeks prior to enrolment
- Planned cardiac surgery within the next 3 months after randomisation

### 5.3 Informed consent

All patients will be informed about background, objectives and risks of the study by the treating physician and will be given the opportunity to discuss all their questions concerning the study.

Only patients who gave written informed consent will be eligible to participate in the trial.

The used informed consent form for the pacemaker implantation and the additional information and consent form for the study are enclosed for review by the ethics board.

The study information and consent form informs the patient additionally about additional, study-related procedures and about anonymized data handling and electronically data processing due to the study.

### 5.4 Data collection and documentation

The study centre receives an Avoid FFS study ring binder and one or several patient folder(s), in which all forms and records for each patient are stored in order to document the study data as well as the informed consent

#### 5.4.1 Patient enrolment

By including a patient an „enrolment form“ with all data will be filled in and a completed copy with a signed patient informed consent form will be archived in the patient´s file.

The following data will be recorded:

- Patient informed consent form
- Anonymized patient identification
  - initials
  - gender
  - date of birth
- Antiarrhythmic medication
- Underlying cardiac disease
- Underlying bradycardia and its symptoms
- Randomisation

#### 5.4.2 Implantation and revision surgery

The patients will receive a licensed pacemaker (Identity ADX DR, XL DR, Victory DR or any dual chamber follow-up model produced by St Jude Medical) and according to randomisation a matching, bipol, CE-certified atrial lead (Tendril 1699 in the study group and Tendril 1388, 1688 or 1788 in the control group), as well as a CE-certified ventricular lead (see section 6).

An implantation form will be filled in for each operation. Even in case of revision surgery without device removal or change an implantation form will document the reasoning for revision.

The following implantation data will be recorded:

- Implantation date
- Device-type, device-id and serial number
- Atrial and ventricular sensing threshold (PSA / pacemaker)
- Atrial and ventricular pacing threshold and lead impedance (PSA / pacemaker)
- Atrial and ventricular signal amplitude (PSA / pacemaker)
- Atrial lead placement
- Adverse events / surgery related events

#### 5.4.3 Follow-Up

Each follow-up visit will be documented by filling in a „follow up“-form. There will be a documentation of even unscheduled pacemaker testing due to arrhythmia, treatments or hospital stays.

For each participant a PHD (Pre Hospital Discharge)-testing of the pacemaker system, as well as follow-up visit in 1 and 3 month(s) after implantation are scheduled.

- **PHD:** prior to hospital Discharge
- **F1-F2:** 1 month und 3 months ± 14 days after primary implantation

##### 5.4.3.1 Follow-up visit und documentation

Following tests will be done and following parameter will be recorded at each follow-up visit:

**PHD**

- Follow-up date
- Current antiarrhythmic medication
- Pacemaker parameters (sensing and pacing thresholds, impedance of the leads etc)
- Keeping records of AF-burden-, mode-switch-, frequency- and sensor histogram, inclusive all stored IEGMs, and deleting the pacemaker storage afterwards
- Far-field sensing test within the control group only (please see 5.4.5)
- Programming according to the study guidelines (please see 5.5)
- Adverse events

**F1 (1 month after implantation ± 14 days)**

- Follow-up date
- Current antiarrhythmic medication
- Pacemaker parameters (sensing and pacing thresholds, impedance of the leads etc)
- Keeping records of AF-burden-, mode-switch-, frequency- and sensor histogram, inclusive all stored IEGMs, and deleting the pacemaker storage afterwards
- Analysing of the mode-switch IEGMs in terms of inappropriate mode-switch episodes. In case of inappropriate mode-switch episodes a participant has reached the primary endpoint. The patient will undergo an individual optimisation of the PVAB according to the far-field sensing test results and stay in the study group until the end of the trial
- Programming of the pacemaker according to the study guidelines
- Any hospital stays since discharge, reason for hospitalisation
- Adverse events

**F2 (3 months after implantation ± 14 days = end of trial)**

- Follow-up date
- Current antiarrhythmic medication
- Pacemaker parameters (sensing and pacing thresholds, impedance of the leads etc)
- Keeping records of AF-burden-, mode-switch-, frequency- and sensor histogram, inclusive all stored IEGMs, and deleting the pacemaker storage afterwards
- Analysing of the mode-switch IEGMs in terms of inappropriate mode-switch episodes. In case of inappropriate mode-switch episodes a participant has reached the primary endpoint. The patient will undergo an individual optimisation of the PVAB according to the far-field sensing test results.
- Final programming of the pacemaker
- Any hospital stays since discharge, reason for hospitalisation
- Adverse events

##### 5.4.3.2 Completed Follow-Up period

After completing the F2-follow-up visit (3 months ± 14 after implantation) of the last participant the trial will end.

Beside the scheduled end of the follow-up there are possibly dropouts ahead of schedule due to withdrawal of the informed consent, changeover to another pacemaker follow-up centre, death of the patient. In all cases there will be a documentation of the dropout-reasons by filling in the from „early end of study“. The following data will be recorded:

- Date of last follow-up
- Reasoning for dropping out

#### 5.4.4 Events / Adverse events

There will be a documentation of every event including date and detailed description on the „event“-form. Furthermore every event has to be classified according to its degree of severity.

- In case of adverse events the sponsor has to be informed within one working day after occurrence
- Additionally in case of serious events the responsible ethics committee and the principal investigator has to be informed directly

#### 5.4.5 Far-field Sensing Test

Only patients within the control group and those of the study group with inappropriate mode switch episodes (stored in the IEGM memory) will undergo a far-field sensing test..

- Hereby intraatrial and intraventricular IEGM (atrial sensing amplification, Vtip-Vring), as well as extended marker and external ECG recordings of the pacemaker programmer are used
- Temporary programmed parametric settings
- Atrial sensing: 0.1 mV, bipolar
- PVAB 60 ms
- AV-time 70ms (ventricular pacing)

An ECG-stripe, recorded with 50 mm/s, has to be printed out. If there are black boxes (representing p-wave sensing within in the refractory period) the interval from the ventricular pacing marker to the P-marker (black box) has to be measured. This interval equals the coupling interval (CI) of the far-field R-wave signal.

The optimised PVAB will be programmed at CI +25ms, but not less than 85 ms.

The testing will be recorded with intrinsic conduction but also under pacing condition. In the course of the study a prolongation of the PVAB according to FFS test results is allowed. But there will be no shortening of the PVAB, only due to relevant clinical reasons.

#### 5.4.6 AV / PV Optimisation

The Choosing of an appropriate test in order to optimise AV / PV are left to each study centre.

### 5.5 Pacemaker programming guidelines

The following parametric settings represent the guidelines for the initial pacemaker programming and should be programmed. Not following the guidelines has to justified and documented..

- DDD- or DDDR-mode
- Atrial sensing: bipolar 0,3 mV
- PVAB within the study group: 60 ms / within the control group: based on the FFS-test (please see 5.4.5)
- AMS switched „on“ (DDIR) with setting the detection-threshold for atrial tachyarrhythmia at 180 min^-1^
- Triggering ECG-recording: AMS switched „on“; all others switched „off“
- Optimising the AV-time in order to enable a intrinsic conduction (via prolongation of the AV-time or via AV-hysteresis)
- Rate-adaptive AV-time: switched „off“

### 5.6 Adverse events (AE)

#### 5.6.1 Guidelines

A documentation of the event in terms of a detailed description, date and time of occurrence, length and course on the case report form is obligatory. Furthermore the event has to be classified in accordance to any association to the study procedure and in accordance to the degree of severity. The acute actions and the following medical treatments have to be documented as well.

Any patient, who face an adverse event, has to be monitored until the end of the event or its final evaluation. The occurrence of new symptoms or new secondary disorders, which have not be present at the inclusion, has also to be valued as an adverse event.

#### 5.6.2 Classification of adverse events

Any adverse event will be classified according to the below defined groups by the principle investigator, investigator or co-investigator. There will be a documentation of the rating on the case report form.

- **Class A:**Event, which is **associated** with a diagnostic assessment, an examination or medical, treatment according to the Avoid FFS study protocol.
- **Class B:**
- Event, which is **possibly associated** with a diagnostic assessment, an examination or medical, treatment according to the Avoid FFS study protocol
- **Class C:**
- Event, which is **not associated** with a diagnostic assessment, an examination or medical, treatment according to the Avoid FFS study protocol.
- **Class D:**Event, whose **association** to a diagnostic assessment, an examination or medical, treatment according to the Avoid FFS study protocol **cannot be evaluated**.

#### 5.6.3 Serious adverse events

All serious adverse events will be classified in according to the degree of severity.

##### 5.6.3.1 Mild adverse events

Events, which may only require basic medical treatment or basic diagnostic assessment and is usually transient

##### 5.6.3.2 Moderate adverse events

Events, which requires medical treatment or diagnostic assessment and is remediable or persist in a tolerable way

##### 5.6.3.3 Serious adverse events

Serious adverse events are any untoward medical occurrence that is life threatening or results in persistent or significant disability/incapacity. Furthermore any adverse events which possibly results in death, requires inpatient hospitalization or prolongation of existing hospitalization, causes congenital anomaly/birth defects or leads to permanent impairment or damage.

In case of serious adverse events the principle investigator, the sponsor and the ethics committee have to be informed immediatly abourt the occurrence.

#### 5.6.4 Reporting of adverse events

Table 1 illustrates the reporting lines and periods in case of adverse envents:

| **Class** | **Degree of severity** | **Who reports?** | **To whom?** | **Within** |
| --- | --- | --- | --- | --- |
| A, B, C, D | mild | Investigator | Sponsor | 3 working days |
|  | moderate |  |  |  |
|  | serious | Investigator | Sponsor,  Principle Investigator,  Ethics committee | 1 working day  Notification by phone in advance |

Table 1: Reporting and reporting lines in case of adverse events

#### 5.6.5 The course of an adverse event

The course of an adverse event will be evaluated along the following criteria at the end of the follow-up period or at the end of the event.

- The adverse event was transient.
- The adverse event is ongoing but persist in a tolerable way
- The adverse event led to a permanent impairment or damage
- The adverse event resulted in death.

### 5.7 Risks

All participants face the general risks associated with a pacemaker implantation and the possible complications in the long-term

**Additional study-related risks:**

none

### 5.8 Advantages

The new atrial lead Tendril 1699 has shown an reducing impact on the far-field R-wave amplitude. Consequently the PVAB can be kept short, which in turn goes along with an even improved atrial sensing. In particular the likelihood of 2:1 lock-in of atrial flutter could be lowered.

Furthermore as no more far-field sensing tests and no more individual adjustments of the PVAB are needed, the use of the lead leads to a time saving in follow-up visits.

## 6 Valid Implants

Within the Avoid FFS Study only CE-certified implants will be used.

Pacemaker: Identity™ ADx DR; Victory**^TM^** DR or follow up model

Atrial lead: Study group: Tendril 1699 / Control group: Tendril 1388, 1688 or 1788

Ventricular lead: CE-certified lead

## 7 Statistical consideration

### 7.1 Randomisation

The randomisation will be performed centre-stratified in groups of 4. Each participating centre will receive its own randomisation in 4 numbered, sealed envelopes. The master randomization list remains in the hand of St. Jude Medical GmbH, Eschborn.

### 7.2 Statistical Methods

Details:

Power = 80%

Groups: 1388/1688/1788 lead vs 1699 lead

Endpoint: % of inappropriate MS due to FFS

When 75 patients are recruited in each group, the lower limit of the observed one-sided 95% confidence interval will be expected to exceed -10% with 80% power when the Standard (1388/1688/1788) proportion, is 5% and the Test (1699) expected proportion, p_T_, is also 5%; results are based on 1000 simulations using the Newcombe-Wilson score method to construct the confidence interval. (Newcombe RG (1988) Interval estimation for the difference between independent proportions: comparison of eleven methods. Statistics in Medicine 17:873-890.)

Hence, assuming a drop-out rate of 10% you need to recruit **168 patients**.

## 8 Stop criteria

In case of adverse events, which are associated with the participation in the study and will harm a single participant, this particular participation won´t be continued. The same also applies to the total study population if harm to the other participants can´t be excluded.

## Ethics and legal aspects

### 9.1 Ethical guidelines

The study will be conducted in conformance with the Declaration of Helsinki, in its valid version of 1996.

Participation in the present trial is entirely voluntary. The patients / participants are free to wwithdraw from the study at any time for any reason and will receive alternative conventional therapy as indicated without any disadvantages.

### 9.2 Patient education/Handout for participants/Patient Informed Consent form

Prior to participating in the study the Investigator will inform the patient orally and in writing about the scope and purpose, and possible risks/ benefits of the study.

The patient´s agreement will be documented by signing the “Patient Informed Consent form”.

In the event of a participant withdrawing from the study his/her existing, already provided data will be deleted and destroyed or will be utilized with the participant’s explicit consent.

### 9.3 Approval by the Ethics Committee

Each investigator (study centre), prior to participation in this study, will obtain medical Ethics Committee approval for the protocol. Involvement of patients will not start without a written approval by the Ethics Committee.

### 9.4 Data security / Access to medical records

Personal data and information are subject to medical confidentiality and to legal requirements by German Data Protection Act (Bundesdatenschutzgesetz BDSG)

Medical or personal data may only be disclosed in an anonymized form, third parties will have access to original medical records.

## 10 Contact

| **St. Jude Medical GmbH**  Helfmann-Park 1  D-65760 Eschborn  Phone: 06196-7711-0  Fax: 06196-7711-247 |
| --- |
| **Study Manager:**  Alexander Hümmer Tel.: 0160 471 08 39  Gabriele Reischl Tel.: 0160 745 64 19  Jörg Scheiner Tel.: 06196 7711 241 |

## 12 Protocol Amendments

## 13 References

1. Fröhlig G, et al.: Bipolar ventricular far-field signals in the atrium. Pacing Clin Electrophysiol 1999; 22: 1604 – 1613

2. Geroux L, et al.: True incidence of far-field R wave oversensing during chronic DDD pacing. Pacing Clin Electrophysiol 2001; 24: 566

3. Kolb C, et al.: Preventricular far-field sensing in the atrial channel of dual chamber pacemakers – An occasional cause of inappropriate mode switch 2004; 10: 231-235

4. Yu CM, et al: Clinical Feasibility Study of Far-Field-Signal Reduction (FSR) Pacing Leads in the Right Atrium. Europace Supplements, presented at Workshop on Cardiac Arrhythmias, Venice, Italy 2005; 17: 15

5. Kolb C, et al.: Management of Far-Field R Wave Sensing for Avoidance of Inappropriate Mode Switch in Dual Chamber Pacemakers. J Cardiovasc Electrophysiol 2006, 17:992-997
